# Face and Mask: “Person” and “subjectivity” in Language and Through Signs

**DOI:** 10.1007/s11196-021-09838-6

**Published:** 2021-04-17

**Authors:** Claudio Paolucci

**Affiliations:** Department of Philosophy and Communication Studies, Via A. Gardino 23, 40122 Bologna, Italy

**Keywords:** Person, Subjectivity, Enunciation, Latour, Benveniste, Linguistics, Semiotics, Guillaume

## Abstract

In this paper, I will deal with the way linguistics and semiotics focus on person and subjectivity in language. I start from two different meanings of the “person” word and from Benveniste and Latour’s theories of enunciation. Later, I deal with the problem of subjectivity in language and I connect it to two different views: Benveniste’s idea that subjectivity is grounded on the “I” and Guillaume’s idea of a primacy of the “he”. Starting from the Iliad and from the semiotic idea of subject, I take side for Guillaume and Latour’s theory: it is the delocutive structure of the “he” which, in language, expresses subjectivity, namely the capacity of the subject to make himself the object of his reflections and of his words.

## Faces and Masks

In this work I will show how the linguistic category of person is connected to the idea of subjectivity. This will be done through a semiotic theory of enunciation grounded on the primacy of the “he”, the category that, according to the linguistic theory of enunciation, used to express the “non-person”.

In order to show the way linguistics and semiotics handle the “personhood” idea, I want to start from the two different Greek and Latin etymologies of the word ‘person’, which both arrive to the “grammatical person” sense, but by means of a totally opposite semantic genealogy. As Létoublon [1] has shown, Greek *prosôpon* primarily means “what is before the eyes”, “face”, “outward appearance” (sense 1). The second meaning of the word is, on the other hand, “physical person” (sense 2), while only much later, via Etruscan influences, *prosôpon* began to mean “mask” (sense 3), “character” (sense 4) and “grammatical person” (sense 5). By contrast, in Latin *persona* primarily and originally means “theatre mask”, “character” (sense 1), and only later “human person, individual” (sense 2) and, finally, “grammatical person” (sense 3).

“Face” VS “Mask”: this is the dichotomy in language connected to personhood. Indeed, the French linguist Emile Benveniste was perfectly aware that ‘I’ and “you” are very special linguistic signs in the sense that, in oral speech, their meaning and reference varies in accordance with who is saying ‘I’ or “you”. This is true of very few other language entities (‘you’, ‘here’, ‘now’) and it is true only in “face to face” communication, where I refer to myself saying “I” and my interlocutor (“you”) can do the very same thing with himself. Enunciation is connected to the Greek sense of “person” here: it is the face of the other (you) in front of me that establishes the “person correlation” (I-you), as Benveniste [2] used to call it. Benveniste remained fascinated, in common with a great many later linguists and philosophers of language, by the “heretical transcendence” of these “ultra-special categories” which change meaning according to who it is saying them, later made famous by the name *embrayeurs*.^1^ Hence a first asymmetry which Benveniste paid much attention to: “I” and “you” are not on the same plane as “he”, as the third person does not change meaning and reference in accordance with who is saying it. For this reason, according to Benveniste, ‘I’ (to an even greater extent that ‘you’) takes linguistic primacy in expressing subjectivity.

As we will see, Benveniste’s first conclusion is true: “I” and “you” are not on the same plane as “he”. His second conclusion, on the other hand, is radically false: “I” has no linguistic primacy in expressing subjectivity. Quite the contrary, as a too frequently forgotten linguist, Gustave Guillaume has shown, “he” has primacy. Since Benveniste used to call the third person ‘non-person’, given that almost all languages express the impersonal form in the third person (“il pleut”, “it rains” etc.), it is an impersonal conception of subjectivity which will be constructed here, from the starting point of the idea of personhood in language.

Indeed, at least one other enunciation model exists, the one which will inspire this work, that is at the heart of Bruno Latour’s book *Enquetes sur les modes d’existence*. For Latour [5], enunciating is first and foremost e-nunciating (*ex-nuncius*), s*ending a nuncius, a messenger who speaks for us.* This messenger can be a different subject or an enunciate, a text or a work produced by the enunciation instance, but “enunciating” does not refer to face to face dialogue at all, but primarily to the operations of *sending*, *mediating* and *delegating*. In Latin “*ex*” means “outside of” and the *nuncius* is “he who takes messages”, “he who lets us know”.^2^ In enunciation, the *nuncius*—the enunciate—is thus *distanced* from he who has sent it (*ex*), it *takes the place of* the enunciating instance and *speaks for it*, even when it is absent. *Distancing, substitution* and *delegation*: according to Latour, enunciation is a theory which has to do with sending and with what is present solely by delegation, but, in actual fact, remains absent. And what remains absent can also be the true intentions of the person enunciating, such as in lying or strategic action, when we want to give an image or message of ours which differs from what we actually are. Enunciation is connected here to the Latin sense of “person”: the *nuncius* we send is a mask that speaks for us. Enunciating is the *act* of sending a *nuncius*, a messenger, a “word bearer” who speaks for us.

This is why the theory of enunciation is thus first and foremost a theory of delegates, a theory of mediators, a theory which enquires into who speaks for us. It is a theory concerning those instances and semio-linguistic entities to whom we delegate our words, who mediate in our relationships with things and represent the background to our perceptions of the world. For this reason, in the place of enunciation there is never solely “the subject”—*ego*—but there are many other “enunciating instances”.

We all know, for example, that society generates discourses, and some say that the body is itself a discourse generator. It is thus no longer a question of ‘subjects’ but rather instances of discourses, *enunciating instances*. The other enunciating instances can thus *not* be amputated from the enunciation, reduced traditionally and mistakenly to the *ego*. [7]

Inside every discourse there are semiotic entities which cannot be reduced to the “I”. For this reason, Benveniste’s theory is extremely unsatisfactory, since it is radically subject-centric and ignores the other enunciating instances reverberating within every language act.

## Subjectivity in Language

Indeed, Benveniste’s place of subjectivity in language was the place of an instance of mediation between the *langue,* namely the social and shared aspect of language, and the *parole,* namely the individual act giving shape to it. Benveniste used to call this “third” instance between the *langue* and the *parole* “enunciation” and used to believe that the subjectivity in the language was precisely a matter of this instance which, for him, was occupied by the “*ego*” (first person). For Benveniste, the subject’s place was thus not that of the individual and subjective act, but rather that of the mediation instance which made us *pass* from the social and shared aspect of language to its individual appropriation within the discourse. For this reason the enunciation instance, which defined subjectivity’s place in language, was defined as “the very act of producing an enunciate” (sentence, statement etc.) which, for Benveniste, occurred by means of placing in discourse a series of virtual categories (*langue*) which could be actualised by means of an individual act (*parole*). It was precisely via this act that, according to Benveniste, the enunciation instance “constituted itself subject”, finding its privileged place precisely in those ultra-special language categories which include certain pronouns (“I”), deixis (“here”) and time indicators (“now”).^3^

Emile Benveniste gave the first formulation of enunciation as a process by which natural language (Saussure's *langue*) is turned into discourse. In between language (conceived of as a paradigmatic system) and speech—already interpreted by Hjelmslev as a syntagmatic system and now specified in its status as discourse — it is indeed necessary to supply mediating structures and to imagine how it is that language as social system can be assumed by the individual realm without as a result being scattered into an infinite number of examples of speech (Saussure's *parole*), outside all scientific cognizance. [8: “Enunciation”]

As we have seen, according to Benveniste, language structures such as “I”, “here” and “now” were the privileged place of expressions of subjectivity because, in contrast to other language categories, their meaning was not immediately understandable within the language forms themselves. Understanding the meaning of “I” or “here”, in fact, requires leaving language behind and considering the concrete context of the discourse at which an instance takes on board the forms of language via an act. “Enunciation” was the name of this act that produces “utterances”. This subjectivity linked to an “I”, expressed by certain privileged language categories which inherently refer outside the language itself, seemed in perfect conformity not solely with the transcendence of a phenomenological “I”, like Husserl’s,^4^ but first and foremost, with certain psychology discoveries which used to connect, at least in temporal terms, the ontogenetic appearance of self-consciousness in infants with the acquisition of language. This is the origin of Benveniste’s enormous success, since he could ground in the dominant discipline of those times—linguistics—a series of approaches borrowed from philosophy and psychology. Benveniste guaranteed these approaches a semio-linguistic scientific character, arguing that the subject founds his reality in the reality of language: de facto, the subject is nothing else than the “ego saying *ego*”.

The “subjectivity” we are discussing here is the capacity of the speaker to posit himself as “subject”. […] Now we hold that that “subjectivity”, whether it is placed in phenomenology or in psychology, as one may wish, is only the emergence in the being of a fundamental property of language. “Ego” is he who says “ego”. That is where we see the foundation of “subjectivity”, which is determined by the linguistic status of “person”. [2: p. 224]

Benveniste’s move fits perfectly with that which founded cognitive semiotics (see [11]): semio-linguistic structures do not express cognitive structures nor are they an extrinsic means for something pre-existing occurring within the body, the mind or the brain (see [12]). Quite the contrary, semio-linguistic structures build an indispensable scaffolding which brings them forth. However, in Benveniste’s terms, his move about subjectivity in language is untenable and quite incompatible with the new knowledge coming from the cognitive sciences. We will thus retain his framework (semio-linguistic structure scaffold and bring forth cognition) but set out their specific features in an alternative form.

In any case, a big part of the success of Benveniste’s enunciation theory is precisely due to the fact that it enabled the subjectivity problem to be posed in a new and original way, even though no enunciation theorist after him ever used the word “subject”, preferring the more neutral expression “instance” at all times. However, it was Benveniste himself who devoted a great many pages—which later became ultra-famous—to the “subjectivity in language” issue. Greimas and Courtés [8: “Enunciation”] noted that “Benveniste's innovative contribution gave rise to a number of exegeses of a metaphysical or psychoanalytic bent, all of them exalting the unexpected reappearance of the subject and permitting the suppression of an ‘anonymous’ conception of language—credited—and discredited—for being a collective system of constraints”.

Let us return, then, to the thesis which made Benveniste’s enunciation theory so successful. According perfectly with the “linguistic turn” he was writing within; his thesis was this:

It is in and through language that man constitutes himself as a subject, because language alone establishes the concept of “ego” in reality, in its reality which is that of the being. [2: p. 224]

Clearly, for Benveniste, the subject derives from the linguistic “I”, but his thesis can certainly not be clearly understood without an understanding of what he meant by “subject” and in what way language can be said to be capable of founding the concept of “ego” in its reality. Fortunately, both are extremely clear in Benveniste’s work.

The “subjectivity” we are discussing here is defined not by the feeling which everyone experiences of being himself (this feeling, to the degree that it can be taken note of, is only a reflection) but as the psychic unity that transcends the totality of the actual experiences it assembles and that makes the permanence of the consciousness. [2: p. 224]

The very close bond between Benveniste’s approach to the problem of subjectivity and psychology and philosophy is very clear, to the extent that Benveniste’s theories were formulated precisely in opposition to those of Sartre in *The Transcendence of the Ego* and Politzer in *Critique of the Foundations of Psychology*.^5^ By “subject”, in fact, Benveniste did not mean the non-linguistic person nor an embodied social actor with his sensitive, emotional component, but rather simply the psychic unit which transcends and brings together the lived experience. Thus, not consciousness, but rather what philosophers call *self-consciousness*, that unit (“I”) which must be able to accompany all lived experience, “synthetic unity of apperception”, as Kant defined it. In classical philosophy, self-consciousness is the never complete *act* with which the subject reflects on himself and *subjectivity consists precisely in the ability to make oneself object of one’s own reflections.*

Consciousness is never solely a matter of intentionality directed at objects but one which is duplicated and reflects on itself. As such it is not solely consciousness but self-consciousness. “I think” and “I think that I think” coincide in such a way that one cannot exist without the other. [14: p. 195]

This is the capacity which, according to Benveniste, derives from the linguistic “I” and, more specifically, from the linguistic idea of “person”, which presides, for example, over the distribution and the correlation of personal pronouns such as “I”, “you” and “he”. The originality of Benveniste’s theory of subjectivity is its *connecting self-consciousness with linguistic person theory*, while thinking that self-consciousness derives: (i) from “the correlation of personality opposing the *I-you* persons to the non-person *he*” [2: p. 204]; (ii) from the “correlation of subjectivity operating within the preceding and opposing *I* to *you*” [2: p. 204], while identifying an “I” which is more profound than any point i) type “I”. In fact, whilst “self-consciousness is possible solely by contrast” and neither *I* nor *you* “can conceive of themselves without the other”, according to Benveniste “ego” always retains a transcendent position and a primacy with respect to a *you* which it itself poses.

“I” is always transcendent with respect to “you”. When I get out of “myself” in order to establish a living relationship with a being, of necessity I encounter or I posit a “you”, who is the only imaginable “person” outside of me. These qualities of internality and transcendence properly belong to “I” and are reversed in “you”. One could thus define “you” as the *non-subjective person*, in contrast to the *subjective person* that “I” represents; and these two “persons” are together opposed to the "non-person" form (= he). [2: p. 201]

If it is the subject which is born with the linguistic “I”, it is because, for Benveniste, no self-consciousness exists without language, or rather, *no identity of the subject exists outside the enunciation instance*, namely outside the discourse situation in which someone enunciates saying “I”. It is in this way that language is capable of founding the concept of ‘ego’ in its reality and the “*reality of language” which Benveniste writes about is precisely enunciation.*

Consciousness of self is only possible if it is experienced by contrast. I use *I* only when I am speaking to someone who will be a you in my address. It is this condition of dialogue that is constitutive of *person*, for it implies that reciprocally I becomes you in the address of the one who in his turn designates himself as *I*. […] As a matter of fact, language is responsible for it in all its parts. *I* posits another person, the one who, being, as he is, completely exterior to “me”, becomes my echo to whom I say *you* and who says *you* to me. [2: pp. 224-5]

For Benveniste, then: i) the subject derives from the linguistic “I”; ii) the linguistic *I* founds subjectivity and, thus, self-consciousness, in the “reality of its being”, which is enunciation; iii) thus, enunciation is the condition of possibility of subjectivity, namely self-consciousness. This is a fundamentally important point.

Benveniste [2: p. 226] says that “if one really thinks about it, one will see that there is no other objective testimony to the identity of the subject except that […] language is so organized that it permits each speaker *to appropriate to himself* an entire language by designating himself as *I*”. Having us believe that anyone who does not accept his view has effectively “not really thought” about something is a rhetorical mechanism which Benveniste makes frequent use of in his writings, at least when he makes statements which he cannot fully motivate, as this one seems to be. But this aspect of his argument strategies aside, if what Benveniste is saying was perhaps tenable in the period he was writing in, accepting it as true today is certainly no straightforward matter to the extent that we now possess a great deal of other “objective testimony to the subject’s identity” which in no way seems to consist in the linguistic *I* and the person category.^6^ Quite the contrary, it would seem that precisely a certain reading of “he” and its delocutive capacity—a term capable of uniting “person” and “non-person”^7^ – is able of identifying the subject’s identity in the capacity a *subject* has of making himself *object* of his own semio-linguistic and cognitive activity.

Thus, subjectivity would not consist in the “ego saying *ego*”, appropriating the language through discourse, as Benveniste argued, but rather in the *subject’s ability to make himself object,* that is the *delocutive status* which makes this duplication of the subject possible and enables him to think of himself as a *person* among other people. The second part of this paper will construct a rigorous semio-linguistic theory on this matter, but, first of all, we must return to Benveniste’s idea that subjectivity is constituted within, and by means of, language and that the subject derives from the linguistic *I*. Is this really the case?

## Semiotics of Subjectivity

As is well known,^8^ the Iliad’s characters certainly do not show formed subjectivity, such as that which we attribute ourselves and which we are used to thinking in terms of—with Benveniste—whose very foundation is in language. But Achilles and Agamemnon certainly have language all the same, just as Ulysses does. However, the status of their subjectivity is profoundly different.

Man in the Iliad does not have a subjectivity like ours. He has no consciousness of his consciousness of the world, he has no inner mental space in which to exert introspection. Volition, planning, initiative are organised without any consciousness and are thus ‘told’ to the individual in the language familiar to him, sometimes with the visual aura of a friend dear to him or a figure of authority or a ‘god’, sometimes simply in a voice. The individual obeys these hallucinatory voices because he cannot not “see” for himself what to do. [15: pp. 101–102]

Perhaps we can put it better. This delocutive “he” (voice, god, friend, etc.) which “tells” the individual his own plans, initiatives and will is this same individual’s “I”, the form of his person and subjectivity. Subjectivity is this “he” of “I” who speaks to “I” and modulates his perspective to the extent that when this mechanism does not function and the subject believes this “he” to be truly a “he”, he is frequently suffering from schizophrenia or hallucinations. This “subject”, this third person “he” speaking to “I” first person thus has a semiotic origin and structure which many of the Iliad’s subjects do not have. For example, when Agamemnon notifies Achilles that he will deprive him of his war booty in the person of the slave Briseis, Achilles’s first reaction is to pull out his dagger and kill Agamemnon. But, after a brief movement, he stops. Certainly, for a modern reader, Achilles' change of mind would be based on a consideration that “revenge is a dish best served cold”, that he will better take revenge and achieve better satisfaction by not killing Agamemnon, but by withdrawing from battle and obliging Agamemnon himself to beg him to return, together with his Myrmidons. Achilles, that is:

(i) must have reflected on himself, positioning his own subject as the very object of his reflections;
(ii) must have been able to build a strategy which delineates a possible world which is alternative to the real world;
(iii) must have bet that this possible world generated by his strategy and his ability to make himself object and act as delocutive subject would ultimately become “the same world” as the real one in future. Ultimately Achilles:
(iv) must have known how to hold back his rage in view of a more profitable future for himself.

But Achilles has *none* of these four abilities, he has none of these four characteristics defining subjectivity. He does not know that he knows, he does not feel that he feels, he does not perceive that he perceives, he cannot make himself object of his reflections, he cannot construct that duplication, that turning back on oneself in which a subject is capable of objectifying himself and duplicating himself to reflect on himself. Achilles cannot subjectify himself and is subject only to the extent that he is subject to the will of the goddess Athena, who, “sent by the heavens” and “visible only to him”,^9^ blocks his arm.^10^

The characters in the Iliad have no subjectivity—only the Gods have this—they have only destiny. They are subjects only in the sense that they are subject to the will of the gods. Where we consider ourselves full subjects capable of taking responsibility for our actions, thinking about our actions and generating strategies in which to objectify ourselves as characters among other characters, Achilles completely lacks this capacity, because he is totally lacking subjectivity.

The narrative conventions as regards the characters are such as to exclude all forms of organisation of the mental space in which we can recognise the mediation of subjectivity. […] As in Homer a mental space in which the world is represented and we take decisions, make plans, assess the various promptings of our instincts and passions to achieve a unitary synthesis is lacking, the same is true of a unitary concept of the person, the ‘I’. [17: pp. 168, 171]

The opposite is true with Ulysses. From the Iliad onwards, he is a character whose specific features and isolation from his fellow adventurers is underlined. What makes Odysseus different from the other heroes is in no way that his is a superior intelligence or owns a better ability to read the situation, but rather “cunning”, a shrewdness (*metis*), his *semiotic capacity to come up with tricks and stratagems*, setting up meaning surfaces capable of lying about the world’s state for the purpose of achieving his goals. *It is this semiotic capacity which gives Ulysses his subjectivity*, that I which the other Homeric heroes are entirely lacking. See, for example, Odysseus’s refusal to punish his maids flirting with their suitors immediately in Canto XX of the Odyssey and compare it with Achilles’s decision not to fight Agamemnon.

Firstly, Odysseus speaks to himself and tries to calm himself down and even goes as far as to objectify himself, using the word ‘you’ to his heart, establishing an intersubjective relationship between locutor and interlocutor entirely interior to the person category, which is presented in the form of a dialogue:

Then he smote upon his breast and rebuked his own heart, saying: ‘Endure, my heart; yea, a baser thing thou once didst bear, on that day when the Cyclops, unrestrained in fury, devoured the mighty men of my company; but still thou didst endure till thy craft found a way for thee forth from out the cave, where thou toughest to die. Endure, my heart; yea, a baser thing thou once didst bear. [21: XX, vv. 18–28]

It is Odysseus who performs the functions which, in the Briseis episode, are attributed to the gods: “He himself meditates, decides, holds back. It is not by chance that we frequently find him being referred to with the epithet ‘divine’ (*theoios*) and very frequently ‘luminous’, in kinship with the Zeus root” [17: p. 176]. It is precisely the subjectivity which Achilles lacked which makes Odysseus similar to the gods, to the extent that Odysseus’s heart obeys its subject just as Achilles did the gods. Here we find the important topography of the instances of enunciation highlighted by Coquet [10]: a judgement instance (subject) considerably detached from a sensitive instance (the heart) which must “bear”, “submit to” and “believe to die” (non-subject). The *subject* (I) speaks to the *non-subject* (you) like a locutor speaks to an interlocutor in a dialogue enunciation context, but a *third instance*—the most fundamental of all—emerges in the third person (he), a delocutive *quasi-subject* who belongs to neither the subject (I) nor the non-subject (you), but rather constitutes the former while it frees the latter: *shrewdness (metis)*. What is *metis,* then, the quasi-subject cited in the third person which supervenes to the ‘I’ (subject), freeing the ‘you’ (non-subject)?

*Metis* is “calculating reason”, “practical rationality”, the structuring form of an *act* capable of establishing the subject and the non-subject. In *Theogony*, Metis is Zeus’s first bride whose son is prophesied to overthrow his father. This is why Zeus swallows Metis when she is pregnant and Metis—“who know more than all the gods and mortal men”^11^—thus lives inside Zeus and advises him, giving him her prophesying ability. Zeus is, in fact, defined *metíeta*, the *metióeis*.^12^ This ability to see possible alternative worlds to the real world is precisely Odysseus’s great capacity together with his strategic thinking, his calculating reason (*metis*) and this semiotic ability to construct deceits, to the extent that Odysseus is frequently called *polymetis*, full of prophesying ability and blessed with a reason which knows many calculations and tricks. These two things—and this is our point—are not unconnected. It is, in fact, his ability to predict the possible behaviour of the Trojans when faced with the horse that enables Odysseus to win the war by deceit and tricks, constructing a signifier surface which can be used for lying. It is his semiotic ability to set up signs hiding objects and taking their places that enables Ulysses to free himself from Polyphemus’s grotto, calculating the wisdom of not killing him right away and reflecting on possible alternative worlds to the real one, in which he himself is one character amongst many others.

Rethinking Polyphemus and other equally famous episodes, such as the building of the horse or that of the singing of the mermaids, it is easy to believe that *metis* is, first and foremost, the ability to offer up an opportune signifier surface to the world, i.e. to take on an outward appearance which does not correspond to one’s interior truth but is opportune in a given situation, suited to a certain plan of action. Odysseus escapes Polyphemus’s grotto and hides, hanging under the belly of a sheep which the blinded giant lets out. The Trojan horse works in the same way, hiding the warriors who will destroy the city under its outward appearance. In the case of the maids […] Odysseus does not let the rage that would betray him get the better of him but pretends to be calm and asleep, maintaining his beggar’s garb with which he protects himself in the midst of his enemies. To be calculating reason, metis must also be simulation and dissimulation, semiotic machine with which to construct appearances. It is a matter of lying, erecting meaning effects, making believe and not letting the truth be known and, at the same time, of establishing an appearance which conceals a secret. [17: pp. 180–181]

Thus *metis* is a semiotic capacity to construct signifier surfaces which substitute objects, conceal them and take their places, saying things about them without these things necessarily being true.^13^ In fact, Umberto Eco [23: p. 17] defined semiotics precisely as the discipline which studies “all that which can be used to lie with”. The semiotic capacity to lie serves to make action effective and strategic. For this to be possible, what is required is not only predicting the behaviour of others (once again *metis* qualities) but first and foremost knowing how to envisage oneself in a multiplicity of possible alternative forms and worlds, acting as another and objectifying oneself as a specific subject of one’s own reflection. From which two different ‘I’s: one who acts and one who watches him act, takes on himself the freedom which he deprives himself of (“endure my heart”) and evaluates his behaviour in various possible contexts. This *duplication of the subject himself for the purposes of effective action,* whether we call it “self-consciousness” or “I”, exists only to the extent that a semiotic capacity to make oneself object of one’s own reflections exists, representing the foundation of subjectivity.

Subjectivity is not born of the linguistic “I” but of the semiotic ability to lie, construct signifier surfaces which build worlds alternative to the real one and placing fictional objects within them. It is the “mask” that makes the “person”, building his/her subjectivity. For this reason, *metis* presents simultaneously Latour’s existence modes of metamorphosis [5: pp. 181–206], technique [5: pp. 207–232] and fiction [5: pp. 233–258]. The I with its self-consciousness ability and its personal structure capable of saying “ego” and “you”, is born of this strategic capacity to construct actions based on the image of the other and his potential responses to the action of the ego. This capacity is not born with language. It is born with semiotics in view of effective action, and verbal language inherits this semiotic ability, constructing a formal apparatus capable of expressing it (formal apparatus of enunciation). The fact that subjectivity develops in children at the end of the secondary intersubjectivity phase [24, 25], frequently prior to the learning of language, is coherent with these ideas. From 9 to 18 months of age, in fact, children develop, firstly, a semiotic ability to deceive others via the non-verbal construction of possible alternative worlds to the real one (pretending to cry, vocalising, pointing, etc.) and equally a reinforced capacity to imagine linked to pretence [26], which leads to the development of pretend play, namely that form of play in which we pretend to be someone else [27]. In this way not only does ontogenetic child development, as it appears in modern cognitive science, detach the subjectivity of the linguistic “I” to connect it up with the imagination, action and the semiotic ability to deceive others, but it also shows that the “I” is the last thing to develop. And that its development depends fundamentally on the intersubjectivity of “you” and the impersonal character of “he”. This completely turns Benveniste’s thesis in which “I” takes primacy over the other linguistic persons on its head. We will look at this in the next section. Cognitive semiotics is thus capable of telling a completely different story on the person notion, on the relationship between the linguistic persons and the subjectivity linked to them, as compared to the pre-cognitive semio-linguistic tradition.

For many reasons, then, Benveniste’s thesis, which sees subjectivity as dependent on the linguistic “I”, is no longer tenable today. The fact that “language is so organized that it permits each speaker to appropriate to himself an entire language by designating himself as I” [2: p. 314] is by no means a sign that subjectivity derives from the linguistic “I”. Quite the contrary, it is a sign of the fact that language expresses that semiotic capacity which renders the subject an instance capable of making itself the object of its own reflections. It is extremely significant that language constructs certain ultra-special language categories such as ‘I’, ‘here’ and ‘now’ which cannot be entirely interpreted within language but require us to put linguistic forms aside, and refer to the concrete situation in which they are used. And within this “formal apparatus of enunciation”, it is the delocutive structure of the third person (“he”) which founds the “I” via the “you”. Benveniste’s hierarchy is thus turned on its head: it is no longer “I-you” versus “he”, as Benveniste argued, but rather “I” versus “I + not-I” (he). We will now see how Gustave Guillaume perfectly expressed this primacy of the delocutive category (he)—which Benveniste mistakenly saw as ‘non-person’—over the person categories (I-you). This leads on to our “impersonal enunciation” thesis [28–30], namely a semiotic theory of enunciation which must be founded on the delocutive category of *he* as extensive linguistic subjectivity term, the union of person *and* non-person. Because only the third person is capable of being I and non-I at the same time and, thus, of expressing that ability to make ourselves object of our reflections which we call “subject”. Gustave Guillaume’s theory of the linguistic person will throw light on this.

## *Persona*. The “I”, “you”, “he” Hierarchy

There is a further ambiguous aspect of Benveniste’s theory which needs to be underlined. As Kleiber noted [4: pp. 34–35], “what characterises the third person and separates it from *I* and *you* is its ability to refer to both individual human beings and to non-human beings and objects”. This idea, already present in the ideas of the Port-Royal grammarians, was one of the principal reasons behind Benveniste’s concept of the third person as a ‘non-person’. There is an ontology, i.e. a specific distribution of humans and non-humans,^14^ behind Benveniste’s decision to juxtapose privately “I-you” (person) against “he” (non-person) and deny ‘he’ any person status whatsoever. And despite the evident fact that, at the grammar level, “he” is *obviously* a linguistic person.

The ability to refer to ‘things’ was not the only argument put forward by Benveniste to justify the divorce between *I/you* and *he* but Benveniste considered it one of the most revealing: “Lastly it is important to be fully aware of the specific features of the ‘third person’ which is the only way in which a *thing* can be predicated verbally”.^15^ [4: p. 36]

Indeed, Maillard [32: p. 60] observed that, in Benveniste’s analysis, there was “a frequent ambiguity in the use of the term ‘person’, which sometimes refers to a purely formal grammatical category and sometimes to a substantial human presence in the enunciate”. It is obviously not a coincidence that a theory whose goal is to found the subjectivity of the self-consciousness on the linguistic capacity of “the ego saying ego” views ‘person’ as exclusively that which can be taken on by human actors (I-you) and ‘non-person’ as what can be taken on by either human actors or non-human actors (he). However, if we take the very same opposition “human” versus “non-human”, it is perfectly clear also in this regards that *he* is by no means a “non-person” (non-human), but rather a “person + non-person” (human + non-human). At this level, too, what is at stake is a participatory opposition involving an extensive term (see [11, 33]) and not a privative opposition involving an absence of the person (see [11]), as Benveniste argued. It is, indeed, precisely Benveniste’s French using “personne” to say “nobody”, as in the expression “Qui as-tu vu?—Personne”.

Thus, there is something extremely valuable and powerful in the very notion of “third person”. The third person is, in fact, a “third element”, a mediator outside the locutor-interlocutor relationship,^16^ just as it is also a “third” in the “human” versus “non-human” opposition. The power specific to the third person, which is an extensive term defining the person (A) + the non-person (non-A), becomes clear if we consider Gustave Guillaume’s point of view [34] in his *Leçons de linguistique*.

## The Primacy of ‘he’

Guillaume opens up an interpretation of the person correlation which is entirely alternative to that of Benveniste (see [4, 35]). Indeed, there is another classic way of presenting the I/you/he triad, which is an alternative to thinking of it as locutor (I), interlocutor (you) and non-locutor (he). In this alternative vision, the third person is not seen as a ‘non-person’ in the sense of being “outside the locutive exchange” [32: p. 61]. This other classic representation of the person correlation consists of conceiving the ‘I’ as he who speaks, or locutor, the ‘you’ as he who is spoken to, or interlocutor, and the ‘he’ as *what is spoken of,* or *delocutive.* It is a conception expressed very clearly since antiquity (see [36]), which has a great exception in the impersonal use of the third person, to which we will return, because in phrases such as “it rains”, the third person is in no way the delocutive object referred to.

However, when the “delocutive” idea comes on stage, everything seems to change. If the first tripartite distribution in locutor, interlocutor and non-locutor was, in fact, entirely internal to the level of enunciation as dialogue locution, in the “locutor-interlocutor-delocutive” triad, the three terms are no longer on the same level:

The definitions of the first two persons (*I* and *you*) are effectively located at the enunciation level while the third person (*he*) is valid at the enunciate level. I and you are enunciation instances, and thus are attributed an enunciation role while the others are instances of the enunciate and thus are attributed a role within the enunciate. [4: § 4]

The enunciate (sentence, product of language) is obviously a fundamental dimension in enunciation theory, since “enunciation” is precisely defined as “the very act of producing the enunciate” (in Benveniste) or “the ability of the enunciate to express the act that produced it” (in Greimas). However, when the level of the enunciate is introduced, many things change radically:

If the level of the enunciate is taken on board it is clear that the opposition between *he* and *I* and *you* no longer works: if ‘he’ is certainly a delocutive and thus “what is spoken of”, *I* and *you* are also delocutives in the same way, namely subjects spoken of, as in saying, for example “I left on Sunday morning” or “You left on Sunday morning”, the locutor is obviously speaking of himself or his interlocutor. This is not a new observation. Apollonius Dyscolus and later, in the early Middle Ages, Priscian, highlighted that persons were not to be analysed solely in relation to the role they play in enunciation, but also in relation to the role they play within the enunciate, i.e. as objects of discourse. [4: § 4]

It is worth noting the short circuit between subject and object: ‘I’, ‘you’ and ‘he’ are simultaneously *subjects spoken of* and *objects of discourse*. Both Benveniste and Guillaume were fully aware of this aspect,^17^ but interpreted it in diametrically different ways. For Benveniste, the fact that “he” represents what is spoken of and works at a different level (enunciate) as compared to that of “I” and “you” (enunciation) is simply a further motive for thinking of it as a *non-person*, privatively opposed to “I” and “you”. By contrast, the fact that the delocutive is also present within “I” and “you” prompts Guillaume to postulate a primacy of “he” over the other two persons, as the third person is the only one of the three to present the delocutive trait exclusively.

Looking at things from closer to - something which has been avoided on this matter - the delocutive person is not absent from any of the three persons. Because we are always speaking of a person which, in the case of the locutive person, is the same person speaking and, in the case of the allocutive person, the same person spoken to. If I say to someone: “You behaved badly”, I’m speaking to him but in speaking to him I am speaking to him of him. What we are witnessing is the simultaneous appearance of the allocutive person and an implicitly conceived delocutive person. It is the same when I say: “I believe this”. It is me speaking but in my words it is me spoken of. Thus the delocutive person is implicitly associated with the locutive person. [34: t10, p. 114]

There is a “third person” in every “I” and in every “you” to the extent that subjectivity in language is defined in its very essence precisely by this joint presence. The fact that the third person is in some way implicitly present also within “I” and “you” means that, within Guillaume’s theory, it takes primacy over the other persons and ends up becoming the most important element in the person correlation specific to the formal apparatus of enunciation:

The third person, underpinning all persons, is the foundation of the enunciation system because, when we speak, we are always speaking of someone (or something). For Guillaume a third person underlies all the other linguistic persons [35: p. 48]

We cannot but be on Guillaume’s side on this point. It is, here, a matter of forcefully asserting this primacy of “he” which takes us in the direction of a theory of *impersonal enunciation*, founded on the category which, for Benveniste, defined the non-person. It is extremely clear that the third person (delocutive) defines a person who expresses the object of the discourse, “what or who is spoken of”. Or perhaps it would be better to say that the third person defines the *subject* of the discourse, as the subject of a discourse is precisely “what is spoken of”, “what underlies” (*sub-iectum*) the language lens, “the main subject of a discourse”?

This short circuit between subject and object, between delocutive third person status and the subject of a discourse, is extremely stimulating in semio-linguistic theory terms. *The third person can be simultaneously subject and object* and this assigns it a decisive role for a semiotic theory of the subjectivity in language, since “subjectivity” is precisely the capacity of a *subject* to make itself *object* of its own reflection. It is thus the third person, with its “person + non-person” participatory structure, which holds subject and object together, neutralizing their opposition itself. This neutral position as regards the “subject-object” opposition is flanked by a corresponding neutral position as regards the “human-non-human” opposition, as “he” is both a “who” and a “what”, whereas “I” and “you” can only be “who”. If we thus adopt Guillaume’s point of view, we see emerge at the level of *all* the linguistic persons that fundamental property which we identified as the subjectivity in language’s trade mark: the subject’s capacity to make itself object of its own reflections, which Benveniste saw as exclusive preserve of “I”, of “ego saying *ego*”.

The distinction between a locutive person speaking, an allocutive person spoken to and a delocutive person spoken of is, certainly, absolutely exact. It could not be otherwise given the straightforward nature of the facts observed. But this distinction, however exact, presents things in an incomplete way. The locutive person is not solely the person speaking. It is also the person who, in speaking, speaks of himself. In the same way, the allocutive person is not solely the person spoken to. It is also the person to whom he is spoken of. Only the third person is truly one, as it is solely the person spoken of. [34: t10, 114]

This is what “subjectivity in language” really is: certainly not Benveniste’s “ego saying *ego*”, but, rather, the *delocutive structure underpinning all the linguistic persons*. This “delocutive form” is to be found in the third person in what we might call its ‘pure’ form. This form defines that duplication inside language in which, even when we say “I” or speak to a “you”, this “I” and this “you” become the object (or subject) of the discourse. Guillaume shows us this with no room for misunderstanding: *it is the delocutive structure of the “he” which, in language, expresses subjectivity,* namely the capacity of each subject to make himself the object of his reflections and of his words.

## Footnotes

1 The expression is not from Benveniste, but comes from Ruwet’s translation of Roman Jakobson’s ‘shifter’ which indicated those language categories which “change reference person in accordance with who is saying them” [3, 4].

2 See [6].

3 Benveniste also indicates some syntactic functions as parts of the formal apparatus of the enunciation (interrogation, intimidation, promise etc.): all these are connected to the first person of the “I” and depend on that.

4 The close bond between Benveniste’s linguistics and phenomenology has been repeatedly highlighted. See, for example, [9, 10].

5 See [13].

6 Psychologists such as Julian Jaynes [15], classical antiquity philologists such as Bruno Snell [16] and semiotics scholars such as Ugo Volli [17] – and this without considering the cognitive science literature which moves practically in a diametrically opposite direction from Benveniste’s—have done much work on this “objective testimony of the subject’s identity”, as Benveniste called it, which seemed absent to him in the period he was writing in.

7 ‘Delocutive’ means that which is outside locution, i.e. what is neither locutor nor interlocutor. We will shortly see how ‘he’, which is both the third person and the non-person, is capable of acting as extensive term within the linguistic category of ‘person’ with its ability to represent all positions. This is a reading inspired by the linguistic theories of Hjelmslev and Guillaume, which *overtly contradicts* Benveniste’s idea, according to which ‘he’ is the exclusive form of the ‘non-person’.

8 See [17, 18].

9 [19: I, 193, 198].

10 Snell comments [16: p. 57]: “The poet had no need here of any ‘apparatus’. Achilles simply controls himself. Athena’s action is a disturbance for us but for Homer she was necessary here. We would expect a ‘decision’, namely reflection and action by Achilles. In Homer, by contrast, man is still not exponent of his decision”. For an important reactivation of linguistic and philosophical ideas connected to *Iliad*, see [20].

11 [22: verses 886–7].

12 See. [18], chapters 3 et 4.

13 [18: pp. 14–5].

14 See [31].

15 [2: p. 230].

16 See [32: p. 61].

17 See, for example, [2: p. 228] and [34: p. 114].

## References

[1] Létoublon Françoise (1994). La personne et ses masques: remarques sur le développement de la notion de personne et sur son étymologie dans l’histoire de la langue grecque. Faits de langue.

[2] Benveniste, Émile. 1966. *Problèmes de linguistique générale*. Paris: Gallimard. English Edition: Benveniste, Émile. 1971. *Problems in General Linguistic,* Transl. Maria Elizabeth Meek, Coral Gables Fl.: Miami University Press.

[3] Jakobson Roman (1963). Essais de linguistique générale.

[4] Kleiber, Georges. 2016. Énonciation et personne ou Quelques moments de la vie d’un couple. In *L’énonciation aujourd’hui. Un concept clé de sciences du langage*, eds. Colas-Blaise, Marion, Perrin, Laurent and Tore, Gian Maria: 33–50. Limoges: Lambert-Lucas.

[5] Latour, Bruno. 2012. *Enquête sur les modes d’existence. Une anthropologie des Modernes*. Paris: La Découverte. English edition: Latour, Bruno. 2013. *An Inquiry into the Modes of Existence. An Anthropology of the Moderns.* Trans. Porter, Catherine. Cambridge (MA): Cambridge University Press.

[6] Pianigiani Ottorino (1907). Vocabolario etimologico della lingua italiana.

[7] Coquet, Jean-Claude. 2016. L’énonciation, fondement de la phénoménologie du langage. In *L’énonciation aujourd’hui. Un concept clé de sciences du langage*, eds. Colas-Blaise, Marion, Perrin, Laurent and Tore, Gian Maria: 295–302. Limoges: Lambert-Lucas.

[8] Greimas, Algirdas J., Jospeh Courtes, and Sémiotique. 1979. Dictionnaire raisonné de la théorie du langage. Paris: Hachette. English Edition: Greimas Algirdas J. and Courtes, Jospeh. . 1982. *Semiotics and Language: An Analytical Dictionary*. Bloomington: Indiana University Press.

[9] Violi, Patrizia. 2007. Lo spazio del soggetto nell’enciclopedia. In *Studi di semiotica interpretativa,* ed. Paolucci, Claudio: 177–202. Milan: Bompiani.

[10] Coquet, Jean-Claude. 2007. *Physis et Logos. Une phénoménologie du langage*. Paris: Presses Universitaires de Vincennes.

[11] Paolucci Claudio (2021). Cognitive Semiotics.

[12] Paolucci, Claudio. 2012. Per una concezione strutturale della cognizione: semiotica e scienze cognitive tra embodiment ed estensione della mente. In *Bioestetica, Bioetica, Biopolitica*, eds. Graziano, Mario and Luverà, Consuelo: 247–276. Messina: Corisco.

[13] Bianco, Giuseppe. 2008. Il professor Benveniste contro gli analogisti. Sulla differenza tra gioco e sacro. *Aut aut*, 337, January/March: 75–92.

[14] Jaspers, Karl T. 1932. *Philosophie*. English edition: Jaspers, Karl. 1969–1971. *Philosophy*. trans. E. B. Ashton, Chicago: University of Chicago Press. Trans. E.B. 1969–1971.

[15] Jaynes Julian (1976). The Origin of Consciousness in the Breakdown of the Bicameral Mind.

[16] Snell, Bruno. 1948. *Die Entdeckung des Geistes. Studien zur Entstehung des europaeischen Denken bei den Grieschen*. 2nd amended edn. Hamburg: Classen Verlag. English edition: Snell, Bruno. 1953. *The discovery of the mind. The Greek origins of European Thought.* Trans. Rosenmeyer, T.G. Cambridge (MA): Harvard University Press.

[17] Volli, Ugo. 1994. La cicatrice di Odisseo. *Il Piccolo Hans*, n. 79–80 (Autunno-Inverno), 1993–4: 162–194.

[18] Detienne, Marcel and Vernant, Jean-Pierre. 1974. *Les ruses de l’intelligence. La* metis *des Grecs*. Paris: Flammarion.

[19] Homer. *The Iliad*. Translated by Robert Fitzgerald, Anchor, 1975.

[20] Piazza, Francesca. 2019. *La parola e la spada. Violenza e linguaggio attraverso l’Iliade*. Bologna: Il Mulino.

[21] Homer. *The Odyssey*. The Harvard Classics. Ed. 1909–14.

[22] Hesiod. *Theogony, Works and Days, Testimonia*. Edited and Translated by Glenn W. Most, Loeb Classical Library 57 (Cambridge, MA & London: Harvard University Press, 2006), LXXXII + 308 pp.

[23] Eco, Umberto, 1975. Trattato di semiotica generale. Milan: Bompiani. English edition, . 1976. *A Theory of Semiotics*. Bloomington: Indiana University Press.

[24] Trevarthen, Colwyn and Hubley, Penelope. 1978. Secondary Intersubjectivity: Confidence, Confiding, and Acts of Meaning in the First Year. In *Action, Gesture and Symbol: The Emergence of Language*, eds. Lock, Andy: 183–22. New York, San Francisco: Academic Press.

[25] Gallagher, Shaun and Hutto, Daniel D. 2008. Understanding others through Primary Interaction and Narrative Practice. In *The Shared Mind: Perspectives on Intersubjectivity*, eds. Zlatev, Jordan, Racine, Timothy, Sinha, Chris and Itkonen, Esa: 17–38. Amsterdam: John Benjamins.

[26] Reddy Vasudevi (2008). How infants know minds.

[27] Paolucci, Claudio. 2019. Social cognition, mindreading and narratives. A cognitive semiotics perspective on narrative practices from early mindreading to Autism Spectrum Disorder. Phenomenology and the Cognitive Sciences, 18 (2), 2019: 375–400.

[28] Paolucci, Claudio. 2017. Prothèses de la subjectivité. L’appareil formel de l’énonciation dans l’audiovisuel. In *Les plis visuels. Réflexivité et énonciation dans l'image*, eds. Dondero, Maria Giulia, Beyaert-Geslin, Anne and Moutat, Audrey: 53–68. Limoges: Lambert-Lucas.

[29] Paolucci, Claudio. 2020. *Persona. Soggettività nel linguaggio e semiotica dell’enunciazione*, Milano, Bompiani. French translation 2020. Liège: Puliège.

[30] Metz, Christian. 1991. *L’ènonciation impersonelle ou le site du film*. Paris: Klincksieck. English edition: Metz, Christian. 2015. *Impersonal enunciation, or the place of film.* Trans. Deane, Cormac. New York: Columbia University Press.

[31] Descola Philippe (2005). *Par*-*delà nature et culture*.

[32] Maillard Michelle (1974). Essai de typologie des substituts diasporiques. Langue française.

[33] Paolucci, Claudio. 2010. *Strutturalismo e interpretazione. Ambizioni per una semiotica minore*. Milan: Bompiani.

[34] Guillaume Gustave (1991). Leçons de linguistique 1943–1944.

[35] Joly André (1994). Eléments pour une théorie générale de la personne. Faits de langue.

[36] Colombat Bernard (1994). Remarques sur le développement de la notion de personne dans l’histoire de la linguistique. La personne.

